# Effect of counselling on health-promoting lifestyle and the quality of life in Iranian middle-aged women: a randomised controlled clinical trial

**DOI:** 10.1186/s12913-019-4176-0

**Published:** 2019-06-03

**Authors:** Vahideh Karimlou, Sakineh Mohammad-Alizadeh Charandabi, Jamileh Malakouti, Mojgan Mirghafourvand

**Affiliations:** 1 Consultation on Midwifery, Midwifery Department, Tabriz University of Medical Sciences, Tabriz, Iran; 2 Midwifery Department, Tabriz University of Medical Sciences, Tabriz, Iran; 3 Social Determinants of Health Research Center, Tabriz University of Medical Sciences, Tabriz, Iran

**Keywords:** Counselling, Health-promoting lifestyle, Quality of life, Middle-aged women

## Abstract

**Background:**

Promotion of healthy lifestyle is an important strategy. This study was conducted to determine the effects of counselling on health-promoting lifestyle and quality of life in middle-aged women.

**Methods:**

This randomised, controlled, clinical trial was conducted on 102 middle-aged women presenting to health centers in Tabriz, Iran, in 2016–17. Using stratified blocking based on age (40–50 and 50–60 age groups) with block sizes of four and six, eligible middle-aged women were randomly allocated to the intervention and control groups. The intervention group received health-promoting lifestyle counselling over three 45-min sessions. The control group received the routine care provided by health centers. The Health Promoting Lifestyle Profile- II (HPLP-II) and quality of life survey (SF-36) were completed in both group before and four and eight weeks after completion of the intervention. Data were analyzed using the independent t-test and the repeated measures analysis of variance (ANOVA).

**Results:**

After adjustment for the baseline values, the repeated measures ANOVA showed that the mean scores of health-promoting lifestyle (adjusted mean difference = 0.91, 95% confidence interval: 0.83 to 0.99, *P* < 0.001) and quality of life (18.2, 15.75 to 20.66, *P* < 0.001) were significantly higher in the intervention compared to the control group after the intervention.

**Conclusion:**

Counselling can improve health-promoting lifestyle and quality of life in middle-aged women.

**Trial registration:**

: IRCT2015122610324N27. Registered 4 February 2016.

## Background

Women play a major role in promoting family and community health. As a result, their physical, psychological, social and spiritual health is of utmost importance [1]. According to a 2016 census, women account for 49.33% of Iran’s population [2]. Adults aged 40–60 are referred to as middle-aged [3]. Middle age is a stage of transition from youth to old age. According to a 2011 census in Iran, 23% of women in the country are middle-aged [2]. Women at this age range experience several health-related events, including biological changes such as the emergence of gray hair and wrinkles, poor eyesight, changes in the state of health, reduced physical ability [4], psychological problems and depression due to the loss of family members or friends [5], physical problems, reduced perceptions of well-being and self-worth, poor quality of life and menopausal transitional period [6].

Menopause is the most critical middle-age event and is followed by many physical and psychological consequences, including osteoporosis, hot flushes, insomnia, cognitive problems, sexual dysfunction [5], increased risk of cardiovascular diseases, diabetes, pulmonary diseases, hypertension, anemia and thyroid disorders, which can be disrupting to life and cause psychological damage [4]. Various studies conducted in different parts of the world have shown the adverse effects of menopause on women’s quality of life [4–7].

Health-promotion and healthy lifestyle activities are the main strategies for health maintenance [8]. According to the WHO, 60% of an individuals’ quality of life and state of health depends on their own behaviours and lifestyle [9]. Walker defines a health-promoting lifestyle as a multidimensional model of self-initiated perceptions and actions that help the continuation and reinforcement of health and self-actualization [10]. Lifestyle comprises the routine daily life activities that affect the individual’s health [11]. According to a meta-analysis based on 15 cohort studies, having a healthy lifestyle is associated with a 66% reduction in all-causes mortality [12]. Lifestyle is important because it affects the quality of life and disease prevention [13].

Quality of life is the individuals’ mental evaluation of their current state of health, the health care he receives and the health-promotion activities he performs, which enable the pursuit of life goals [14]. Although women live longer than men, their quality of health is generally poorer than men’s; meanwhile, it is women’s healthiness that provides the energy required in the family and ensures the development of a dynamic and healthy community [15]. The main health challenge of the twenty-first century is to improve the quality of life and promote health by empowering people to have greater control over their own health [16]. Counselling is one of the best approaches for empowering people in this area [17].

Counselling is a helpful tool in which one is trained in the principles and practices of selecting, planning, and continuing a reasonable and successful life [18]. Studies have investigated the effect of counselling on health-promoting lifestyle in mothers with gestational diabetes [19], older adults [20] and adults at increased risk of coronary heart disease [21]; however, no studies of which we are aware have specifically addressed the effect of counselling on health-promoting behaviours and quality of life in women in the middle age group. Considering that there are several psychological, social, and biological factors responsible for the change in life and lifestyle of middle-aged women [3], and also given the role of women’s health in public health promotion, this group of the society needs further emphasis in its different periods of life. The present study assessed the effects of counselling on health-promoting lifestyle (as the primary outcome) and quality of life (as the secondary outcome) in middle-aged women.

## Methods

CONSORT guidelines were adhered for reporting of this trial.

### Study design and participants

This randomised, controlled, clinical trial with two parallel arms recruited 102 middle-aged women presenting to health centers in Tabriz, Iran, in 2016–17. The study was conducted on women aged 40–60 who had a low HPLP-II scores (≤2.5) [22, 23] and were willing to participate and could offer a contact number for follow-up. The exclusion criteria consisted of a history of physical illness (based on the self-reports), recent adverse life events, smoking, alcohol and substance abuse (exerting physical and mental effects), extreme poverty and a history of participation in lifestyle-promoting classes according to the participants’ expression.

G*Power 3.1.2 was used for calculating the sample size based on health-promoting behaviours. Health-promoting behaviours have formerly been described in Iran in a study by Enjezab et al. (2012) conducted on middle-aged women; in view of these results and to account for the largest standard deviation of the subscales of health-promoting lifestyle in middle-aged women [24] and considering m_1_ = 14.2, the chance of a 20% increase in the health responsibility score (m_2_ = 57.2), sd_1_ = sd_2_ = 68.0, test power = 90% and α =0.05, the sample size was determined as 46 per group, but was increased to 51 to account for a potential withdrawal rate of 10%.

### Sampling

Sampling began upon the approval of the ethics committee of Tabriz University of Medical Sciences (1394.900.TBZMED.REC) and the registration of the study at the Iranian Registry of Clinical Trials (code: IRCT2015122610324N27). Convenience sampling was first performed across one-third of the 70 health centers of Tabriz, which were randomly selected from different socio-economic regions of the city. Health centers have been established in Iran to provide free services to the general public. These state-funded centers are run by health care professionals such as midwives, family health experts, nutritionists, psychologists and physicians. Based on a national census, all Iranian households have health records in these centers, which provide services to pregnant women, adolescents, middle-aged people and older adults of both genders. The people visiting these centers receive services free of charge. There are 70 health centers in Tabriz. Since premenopausal women have medical records at health centers, the patient records at the select centers were reviewed and a list of women under age 60 (including their name and contact number) was prepared. These women were contacted over the phone and the eligible ones were invited to visit the health centers. During their visit, the women who were willing to participate were asked to submit an informed written consent form. A socio-demographic questionnaire and the Health Promoting Lifestyle Profile- II (HPLP-II) and the Short-Form Health Survey (SF-36) were completed through interviews with the participants. The women with low HPLP-II scores (≤2.5) [22, 23] entered the study.

### Randomization

Middle-aged women who had completed the pretest questionnaires were randomly assigned to the intervention (counselling) and control groups using a computerized table of random numbers and stratified block randomization depending on the participant’s age (age groups 40 to 50 or 50 to 60) with block sizes of four and six. Blocking was performed by a person not involved in the sampling. The type of intervention to be given was written on a piece of paper and placed in opaque envelopes numbered consecutively (allocation concealment). The envelopes were given out by the order in which the participants entered the study and the type of intervention was thus identified.

### Intervention

The first author (V. K.) provided counselling to the intervention group. An assistant researcher with a master’s degree in psychology held stress management and interpersonal relationship training sessions for the intervention group and provided counselling on these subjects. The intervention group received three 45-min sessions of group counselling every week to learn about the health-promoting behaviours at the conference room of the select health centers. Each group was composed of a minimum of five and a maximum of nine women. Counselling principles and techniques were used for effective communication with the women, and the counselling sessions had a respectful and friendly atmosphere that reinforced self-confidence and facilitated participation in group discussions. The issues discussed in the counselling sessions included proper nutrition, physical activity, stress management, improving interpersonal relationship techniques, strategies for improving spiritual growth and health responsibility. On the first day of counselling, after the groups were introduced to each other and the researcher’s phone number was distributed among the participants to contact for asking their questions, counselling was provided on nutrition and health responsibility; at the end of this session, educational booklets were distributed to the participants. The second session was concerned with physical activities and spiritual training. Counselling on interpersonal relationships and stress management was provided in the third session. Each session ended with a Q&A. A total of 96% of the participants attended all the counselling sessions, and the absentees were only absent in one session, although one person was absent in two sessions and another one in three; later, the absentees received individual counselling to compensate for the missed sessions. The control group received the routine care provided by health centers (including blood pressure measurement, screening for diseases such as hypertension, neurological and psychiatric diseases, diabetes, etc., and a visit by the resident physician). The control group received the educational booklet on lifestyle after the counselling sessions were over and the posttest questionnaires were completed. The content of booklet was similar to topics discussed in the counselling sessions.

### Data collection tools

A socio-demographic questionnaire, the Persian versions of HPLP-II [24] and the short-form quality of life survey (SF-36) [25] were used in this study. The HPLP-II and SF-36 were completed in both group before and four and eight weeks after completion of the intervention through interview with participants by first author (V. K.) in health centers.

The HPLP-II was used to collect data on health-promoting lifestyles. The original version of this questionnaire was developed by Walker et al. (1987) and contains 52 items and can be used for all age groups [10]. Its content validity has been confirmed in Iran for middle-aged women by Enjezab et al., who increased the number of items to 70 and reported a Cronbach’s alpha of 0.92 [24]. The HPLP-II contains 12 items on nutrition, seven on physical activity, nine on spiritual growth, 23 on health responsibility, six on stress management and 13 for interpersonal relations. Each item is scored on a four-point Likert scale from 1 to 4, including *never* (1 point), *sometimes* (2 points), *often* (3 points) and *routinely* (4 points).

The 36-item survey (SF-36) assesses people’s health status. Montazeri et al. (2005) assessed and confirmed its validity in Iran [25]. The items of this questionnaire measure eight main constructs related to personal health, including four that measure physical health and four that measure psychological health. Each item is scored from 0 to 100, and the sum of the scores of the items in each construct shows the total score for that construct. Lower scores indicate a poorer quality of life and higher scores indicate a better quality of life.

In the present study, the content and face validity assessment methods were used to determine the validity of the socio-demographic questionnaire. The reliability of the HPLP-II and SF-36 was confirmed using a test-retest on 20 people. The Cronbach’s alpha was calculated as 0.947 for HPLP-II and 0.946 for SF-36. The Intra-class Correlation Coefficient (95% Confidence Interval) was calculated as 0.952 (0.878 to 0.981) for HPLP-II and 0.921 (0.800 to 0.969) for SF-36.

### Data analysis

Data were analyzed in SPSS-21. The normal distribution of the quantitative variables was determined using the K-S test. To compare the groups in terms of socio-demographic characteristics, the independent t-test, the Chi-square test, the Chi-square test for trend and Fisher’s exact test were used, and the groups were compared in terms of their mean health-promoting lifestyle and quality of life scores using the independent t-test before the intervention and using the repeated measures analysis of variance (ANOVA) after the intervention. *P* < 0.05 was set as the level of statistical significance. All the analyses were performed based on the intention-to-treat.

## Results

**Fig. 1:**
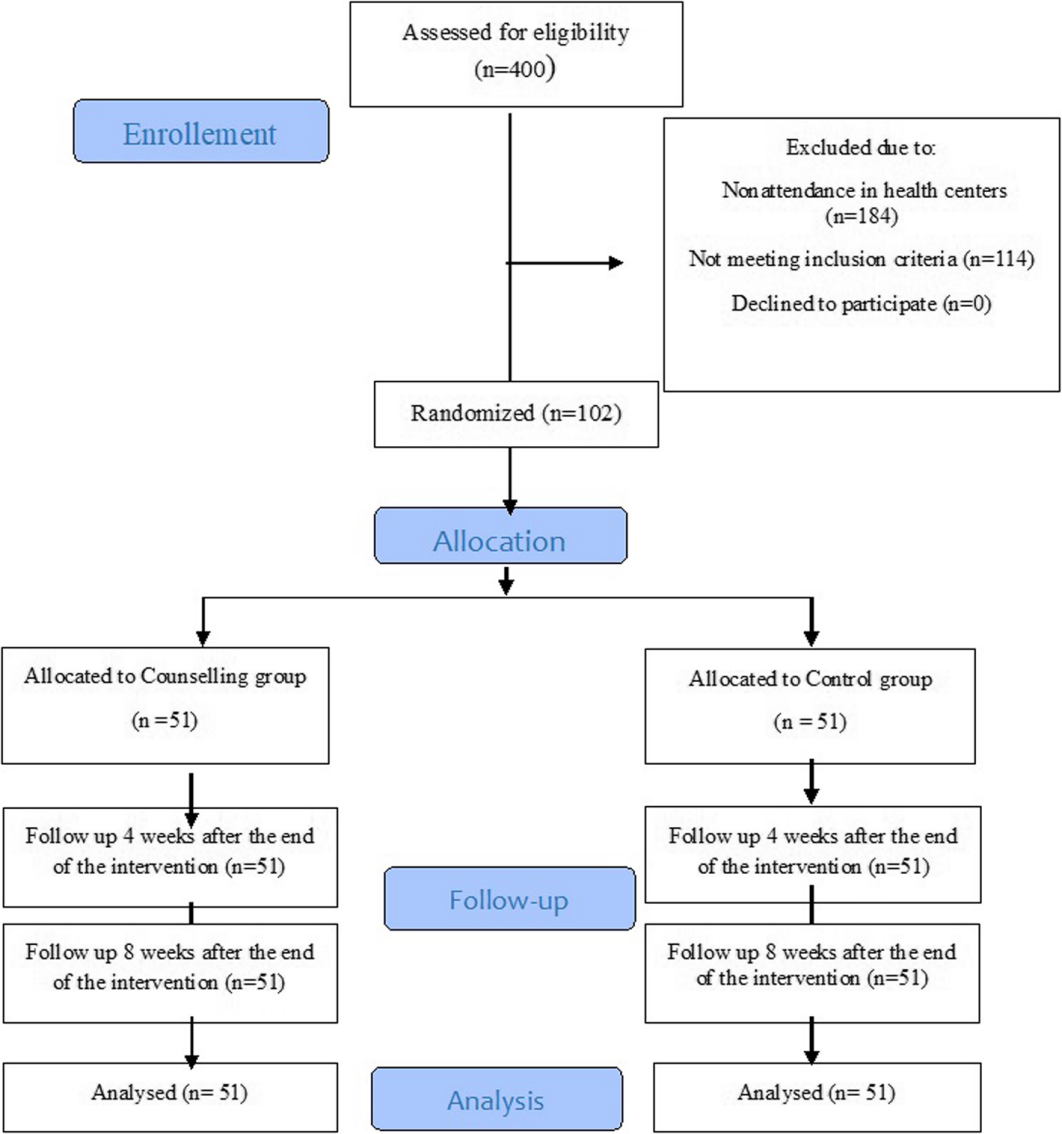
Flowchart of the study

The present study began in February 2016 and ended in September 2017. A total of 400 middle-aged women were assessed in terms of socio-demographic characteristics, including 102 eligible candidates who were randomised into an intervention and a control group. The participants were followed up until the end of the study and there were no cases of withdrawal. Figure 1 indicates the study’s flow diagram based on CONSORT guidelines.

**Fig. 2:**
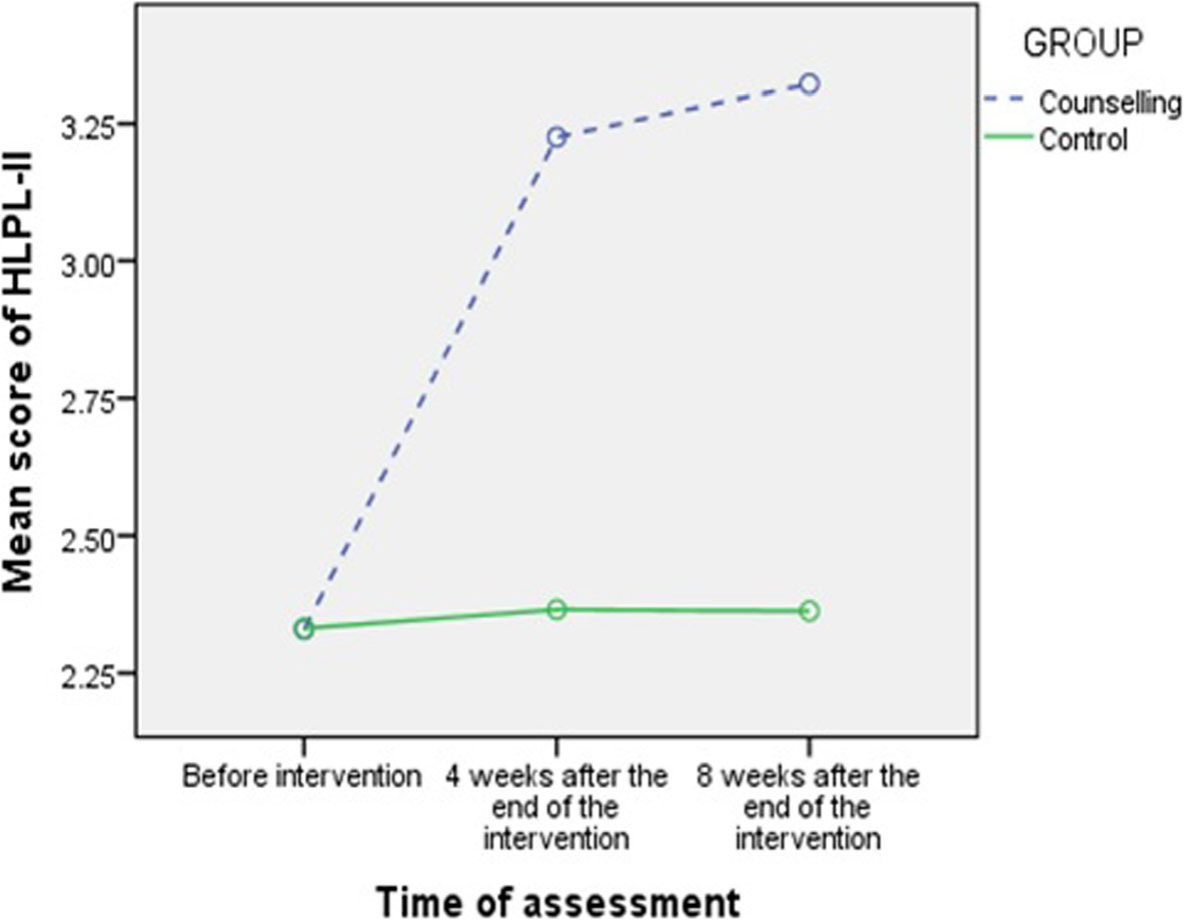
Comparison of health promoting lifestyle score at different time-points by the study groups

Table 1 presents the socio-demographic characteristics of the participants by group. The mean (SD) of health promoting lifestyle was 2.33 (0.20) in the counselling group and 2.33 (0.22) in the control before the intervention. Four weeks after completion of the intervention, average (SD) score of health promoting lifestyle was 3.22 (0.26) in the counselling group and 2.36 (0.26) in the control, and eight weeks after completion of the intervention, 3.32 (0.25) in the counselling group and 2.36 (0.27) in the control. The repeated measures ANOVA (after adjusting for the baseline values) showed significantly higher mean health promoting lifestyle score (Adjusted difference = 0.91, 95% confidence interval: 0.83 to 0.99, *P* < 0.001) (Table 2, Fig. 2).

**Table 1:** Socio-demographic characteristics of the participants

Socio-demographic characteristics	Counselling group	Control group	*P*
	*n* = 51	*n* = 51	
	*n* (%)	*n* (%)	
Age (Year)^e^	46.6 (5.3)	47.2 (5)	0.578^a^
Body Mass Index (kg\m^2^)^e^	28.7 (4.2)	21 (4.9)	0.460^a^
Marriage age^e^	20.5 (4)	21 (4.9)	0.540^a^
Level of Education			0.313^b^
Illiterate	4 (7.8)	5 (9.8)	
Primary school	12 (23.5)	5 (9.8)	
Secondary school	11 (21.6)	12 (23.5)	
High School	4 (7.8)	5 (9.8)	
Diploma	18 (35.3)	21 (41.2)	
University	2 (3.9)	3 (5.9)	
Participant’s job			0.567^c^
Housewife	43 (84.3)	45 (88.2)	
Employed	8 (15.7)	6 (11.8)	
Number of children			0.897^c^
1–2	28 (54.9)	25 (50.0)	
3–5	20 (39.2)	22 (44.0)	
Higher than 5	3 (5.9)	3 (6.0)	
Marital status			1.000^c^
Single	3 (5.9)	4 (7.8)	
Married	48 (94.1)	47 (92.2)	
Husband’s job			0.611^d^
Employed	17 (34.0)	14 (28.0)	
Worker	6 (12.0)	6 (12.0)	
Shopkeeper	6 (12.0)	10 (20.0)	
Freelancer	15 (30.0)	8 (16.0)	
Other	6 (12.0)	12 (24.0)	
Spouse’s education level			0.689^b^
Illiterate	2 (4.0)	4 (7.8)	
Secondary school	8 (16.0)	4 (7.8)	
Secondary school	6 (12.0)	9 (17.6)	
High School	3 (6.0)	3 (5.9)	
Diploma	16 (32.0)	21 (42.2)	
University	15 (30.0)	10 (19.6)	
Family income			0.866^b^
Enough	6 (11.8)	10 (19.6)	
Quite enough	37 (72.5)	30 (58.8)	
Inadequate	8 (15.7)	11 (21.6)	
Religious meetings			1.000^c^
Yes	36 (70.6)	36 (70,6)	
No	15 (29.4)	15 (29.4)	
Marital Satisfaction			0.136^c^
Totally satisfied	11 (22.4)	18 (36.0)	
Fairly satisfied	28 (57.1)	26 (52.0)	
Dissatisfied	4 (8.2)	2 (4.0)	
No idea	6 (12.2)	4 (8.0)	

The data were presented by number (percent) unless it has been shown with ^e^that indicate mean (Standard deviation)

^a^Independent t-test ^b^Chi-square by trend ^c^Fishers exact test ^d^Chi-square tes

**Table 2:** Comparison of health promoting lifestyle score at different time-points by the study groups

Time of sassement	Counselling group	Control group	Time effect	Time & group effect
	*n* = 51	*n* = 51		
	Mean (SD^a^)	Mean (SD^a^)		
			p	p
Before intervention	2.33 (0.20)	2.33 (0.22)	0.331	< 0.001
4 weeks after the end of the intervention	3.22 (0.26)	2.36 (0.26)		
8 weeks after the end of the intervention	3.32 (0.25)	2.36 (0.27)		
Comparison between groups	Adjusted mean difference (95% CI^b^)		*p-value*	
Counselling with Control	0.91 (0.83 to 0.99)		< 0.001	

The results is according to repeated measure ANOVA

^a^Standard Deviation ^b^95% Confidence Interval

**Fig. 3:**
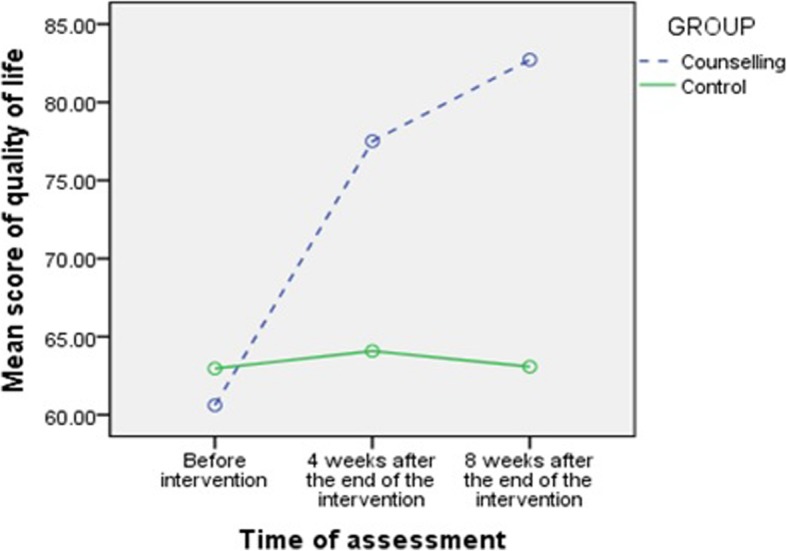
Comparison of quality of life score at different time-points by the study groups

Before the intervention, the mean (SD) score of quality of life was 60.88 (17.04) in the counselling group and 62.60 (15.42) in the control group. Four weeks after completion of the intervention, mean (SD) of quality of life was 77.21 (11.37) in the counselling group and 63.11 (14.63) in the control, and eight weeks after completion of the intervention, 82.53 (8.34) in the counselling group and 62.44 (13.17) in the control. The repeated measures ANOVA (after adjusting for the baseline values) showed significantly higher mean quality of life score (18.21, 15.75 to 20.66, *P* < 0.001) in the counselling group compared to the controls (Table 3, Fig. 3).

**Table 3:** Comparison of quality of life score at different time-points by the study groups

Time of assessment	Counselling group	Control group	Time effect	Time & group effect
	*n* = 51	*n* = 51		
	Mean (SD^a^)	Mean (SD^a^)	p	p
Before intervention	60.88 (17.04)	62.69 (15.42)	< 0.001	< 0.001
4 weeks after the end of the intervention	77.21 (11.37)	63.11 (14.63)		
8 weeks after the end of the intervention	82.53 (8.34)	62.44 (13.17)		
Comparison Between Groups	Adjusted mean difference (95% CI^b^)		*p*-value	
Counselling With Control	18.21 (15.75 to 20.66)		< 0.001	

The results is according to repeated measure ANOVA

^a^Standard Deviation ^b^95% Confidence Interval

## Discussion

The present study is the first to evaluate the effect of counselling on health-promoting lifestyle and quality of life in middle-aged women in Iran. In this study, counselling improved health-promoting behaviours and quality of life in middle-aged women. Given the lack of similar studies on the effect of counselling on health-promoting lifestyle and quality of life in middle-aged women, studies that had assessed the effect of counselling and education on other groups will be used for this discussion.

In a systematic review, the effect of face-to-face interventions for promoting of physical activity in adults aged 16 years and above were compared with a control exposed to placebo or no or minimal intervention. A total of 10 studies that were all conducted in high-income countries were included in this systematic review. The effect of intervention on self-reported physical activity was positive but not sustained in three studies at 24 months [19].

The effect of counselling on health promoting behaviours in pregnancy and postpartum periods has been assessed in two studies in Iran [20, 26]. In both studies, HPLP-II has been used for measuring the health-promoting lifestyle. In a randomised controlled trial on 60 mothers with gestational diabetes, the intervention group received 5 counselling sessions and the control group received no intervention. Post intervention assessment was conducted one week after the end of intervention. The results showed that HPLP-II score were significantly higher in the intervention group than control group [20]. In another randomised controlled trial on 112 primiparous mothers, the intervention group received health promoting counselling in three weekly sessions and the control group received no intervention. The results indicated that counselling leads to improve of health promoting behaviours in mothers [26]. Also, in another before-after trial in Iran on 200 older adults (86% women and 24% men), the effect of educational intervention on some healthy behaviour were assessed. Duration of intervention was nine months. The interventions included home visits, referral to physicians, educational pamphlets, a general meeting- question and answer session and attending at exercise sessions. The educational fields were about mental health, nutrition and exercise. The results indicated that aerobic exercises and life satisfaction improved significantly in women after the intervention, however, no positive effect of intervention was observed in men [21].

Similar studies have been conducted in other countries on patients at increased risk of coronary heart disease [27] or hypertensive patients [28]. In both studies, counseling has been provided based on the stage of change model. Steptoe et al. conducted a cluster randomised controlled trial in London-United Kingdom on 883 men and women at increased risk of coronary heart disease (316 intervention and 567 control). Brief behavioural counselling based on the stage of change model was provided to decrease smoking and dietary fat intake and to promote physical activity. The control group received no intervention. The variables of diet, exercise and smoking habits were measured at 4 and 12 months. They indicated that counseling led to improvement in health behaviour including nutrition and exercise [27]. In another controlled trial, the hypertensive patients were randomly assigned into two intervention groups and a control group. Intervention groups received a high or low level of counselling. The intervention groups were assessed every four weeks for eighteen weeks. The low intervention group received one practice appointment and five telephone counselling while the high intervention group received six appointments. The counselling were provided to reduce alcohol consumption, dietary fat and salt intake and weight; cease smoking; and promote physical activity. The intervention groups indicated significant decreases in alcohol and salt intake compared with controls [28]. Also, in a study conducted in Copenhagen by Nisbeth et al., effectiveness of counselling over 1 year on changes in lifestyle and coronary heart disease risk factors were assessed. In this trial, 152 male aged 25–45 years participated. They were randomised into an intervention group and a control group. The results showed that counselling programs increased regular exercise in the intervention group [29].

Based on the results of the present study and the above mentioned studies, counseling and training are found to have a significant role in promoting healthy lifestyles. Health-promoting behaviours are important because they can improve quality of life. In a study conducted in China on people older than 50, health-promoting behaviours improved quality of life [30]. In another study, the positive effects of physical activity on the quality of life in obese adults were confirmed [31]. Considering the positive effect of counseling in promoting of healthy lifestyle and the quality of life, health providers should be well advised to provide counseling sessions for women of all ages. It seems that such interventions should be continued for sustainable improvement in lifestyle. This study used face to face counseling sessions to address women’s concerns regarding healthy lifestyle. Further research on different education and counseling methods that require less health care resources such as technology-based counseling or written education materials is recommended.

The strengths of the present study include observing all the principles of performing clinical trials, including random allocation and allocation concealment to prevent selection bias. Self-reported nature of assessment is a limitation of this study as well as we do not know whether perceptions of healthy lifestyle on a 4 point likert may not translate into real, long term behaviour and/or sustained quality of life. Also, given that the counselling mapped on to key constructs tested by the HPLP-II, it might be some degree of social desirability bias in the primary outcome. Short length of study to assess ongoing behaviour change is other limitation. Another limitation is that we cannot generalize the results to the women who have a moderate or high HPLP-II scores (> 2.5), because we studied participants with a low HPLP-II scores (≤2.5). It is imperative for counselors to have an active presence in health centers and health centers in order to better introduce the public to counselling and its benefits for their quality of life.

## Conclusion

Counselling can promote a healthier lifestyle and improve the quality of life and overall health in middle-aged women. Of course, these findings were sustained over two follow-up visits implying that the counselling has potential long term benefits in only a few sessions. Given the importance of women’s role in the community and in view of the present findings, promoting healthy lifestyle in women with the assistance of expert counselors in health centers appears promising for aiding a healthy and dynamic community.

## Data Availability

Datasets used and analyzed during this study are available from the corresponding author on reasonable request.

## Abbreviations

ANOVA: Analysis of variance
HPLP-II: Health-promoting lifestyle profile-II
K-S: Kolmogorov-Smirnov
Q&A: Questions and Answers
SD: Standard Deviation
SF-36: Short Form-36
WHO: World Health Organization
